# A Broad-Spectrum Horizontal Transfer Inhibitor Prevents Transmission of Plasmids Carrying Multiple Antibiotic Resistance Genes

**DOI:** 10.1155/2024/7063673

**Published:** 2024-05-30

**Authors:** Yuqian Jia, Zhiwan Zheng, Bingqing Yang, Haijie Zhang, Zhiqiang Wang, Yuan Liu

**Affiliations:** ^1^ Jiangsu Co-innovation Center for Prevention and Control of Important Animal Infectious Diseases and Zoonoses College of Veterinary Medicine Yangzhou University YangzhouChina; ^2^ Department of Pathogenic Biology West China School of Basic Medical Sciences and Forensic Medicine Sichuan University ChengduChina; ^3^ Joint International Research Laboratory of Agriculture and Agri-Product Safety The Ministry of Education of China Yangzhou University YangzhouChina; ^4^ Institute of Comparative Medicine Yangzhou University YangzhouChina

## Abstract

The dissemination of antimicrobial resistance (AMR) severely degrades the performance of antibiotics and constantly paralyzes the global health system. In particular, plasmid-mediated transfer of antibiotic resistance genes (ARGs) across bacteria is recognized as the primary driver. Therefore, antiplasmid transfer approaches are urgently warranted to resolve this intractable problem. Herein, we demonstrated the potential of azidothymidine (AZT), an FDA-approved anti-HIV drug, as a broad-spectrum horizontal transfer inhibitor to effectively prevent the transmission of multiple ARGs, including *mcr-1*, *bla*_NDM−5_, and *tet*(X4), both *in vitro* and *in vivo*. It was also noteworthy that the inhibitory effect of AZT was proved to be valid within and across bacterial genera under different mating conditions. Mechanistic studies revealed that AZT dissipated bacterial proton motive force, which was indispensable for ATP synthesis and flagellar motility. In addition, AZT downregulated bacterial secretion systems involving general and type IV secretion systems (T4SS). Furthermore, the thymidine kinase, which is associated with DNA synthesis, turned out to be the potential target of AZT. Collectively, our work demonstrates the broad inhibitory effect of AZT in preventing ARGs transmission, opening new horizons for controlling AMR.

## 1. Introduction

Antimicrobial resistance (AMR) poses a significant risk to public health at alarming rates. The ubiquitous overuse of antibiotics, especially in animal husbandry, is a leading cause of AMR in humans, animals, and environments [1, 2]. For example, as a vast reservoir of antibiotic resistance genes (ARGs), the mariculture system could give rise to the development of multidrug-resistant (MDR) pathogens [3]. Undoubtedly, the rapid horizontal transmission of ARGs between different bacterial cells accounts for the accumulating AMR crisis [4, 5]. Conjugation is generally considered the main mechanism of horizontal gene transfer (HGT) [6, 7], which permits the transfer of genetic information between bacterial cells [8]. It is a process of DNA transfer from the donor to the recipient bacteria through a pilin bridge across the cell membrane [9]. Another HGT pathway is transformation, which involves the uptake of extracellular DNA fragments and the incorporation of genetic elements by competent cells [10].

Given the rampant evolution and prevalence of AMR, potential inhibitors against ARGs transfer are urgently required. For instance, the synthetic fatty acid 2-hexadecynoic acid inhibited the conjugative transfer of the model plasmids in controlled water microcosms [11]. Similarly, our previous study demonstrated that melatonin, known as a neurohormone that facilitates sleep, prevented conjugative transfer of *mcr*-positive plasmids by disrupting the proton motive force (PMF) [12]. Despite the identification of several conjugation inhibitors, no compounds have been allowed in the clinic owing to their pharmacological and toxic properties. Meanwhile, these conjugative transfer inhibitors are only active against specific plasmid types or plasmids carrying specific ARGs. Thus, we speculated whether there are some compounds with broad-spectrum inhibitory effects on the transmission of multiple resistance genes.

Azidothymidine (AZT), an FDA-approved nonantibiotic compound, has been used for human immunodeficiency virus (HIV) treatment. AZT is a prodrug activated within the cell by a thymidine kinase-mediated phosphorylation event, producing AZT-triphosphate, the active ingredient in DNA strand termination [13]. In addition to the treatment of acquired immune deficiency syndrome (AIDS), our previous study also indicated that AZT decreased *tet*(X)-mediated bacterial resistance to tigecycline in *Escherichia coli* [14]. However, the potential of AZT as a novel horizontal transfer inhibitor remains unexploited.

In this study, we comprehensively investigated the effect of AZT on the transmission of multiple plasmids carrying clinically relevant ARGs within and across bacterial genera. Furthermore, we elucidated the potential mechanisms of AZT behind its broad inhibitory effect on HGT. Finally, we conducted a murine model to confirm the feasibility of an AZT-based antiplasmids strategy.

## 2. Materials and Methods

### 2.1. Bacterial Strains and Mating System

Different clinical strains containing *mcr-1*, *bla*_NDM−5_, and *tet*(X4)-bearing plasmids were selected as donor bacteria, while *E. coli* EC600 and *K. pneumoniae* YZ6 were chosen as recipient bacteria for the conjugation system. The detailed information on strains and plasmids used in this study was shown in Table S1. The conjugation assay was conducted as follows: a single bacterial colony was selected into 1 mL antibiotic-containing LB broth and cultured to achieve OD_600_ of 0.5. After centrifugation for 10 min, PBS was replenished, and different concentrations of AZT were added to the mating system while the donor and recipient bacteria were mixed in a ratio of 1 : 1. After 12 hr culture in the horizontal shaker at 37°C, the transconjugants and recipient bacteria were selected in a plate containing corresponding antibiotics. Specifically, the transconjugants were screened with 2 *μ*g/mL colistin and 300 *μ*g/mL rifampicin when *E. coli* LD93-1 and *E. coli* LD67-1 as donor bacteria and *E. coli* EC600 as recipient [15]. 2 *μ*g/mL meropenem and 300 *μ*g/mL rifampicin were applied when *E. coli* L65 and *K. pneumoniae* C12 were used as donor bacteria [16]. The transconjugants from the mating system using *E. coli* (RS3-1 and RF2-1) [17] and *Proteus* (TCP70-4 and ICP17-4) [18] as donor and *E. coli* EC600 as recipient bacteria were selected using 4 *μ*g/mL tigecycline and 300 *μ*g/mL rifampicin. Meanwhile, plate agar containing 4 *μ*g/mL tigecycline and 100 *μ*g/mL hygromycin was used to screen the transconjugants when *tet*(X4)-bearing plasmids transferred from *E. coli* to *K. pneumoniae* YZ6. The conjugation frequency was calculated using the number of transconjugants divided by the recipient bacteria.

Minimum inhibitory concentration (MIC) determination was conducted using the broth microdilution for antimicrobial susceptibility testing according to the EUCAST clinical breakpoints methods [19]. The results were recorded as the lowest concentration of the antimicrobial agent, which inhibited the visible growth of the bacteria. Also, PCR verification of the *mcr-1* [20], *bla*_NDM−5 (16)_, and *tet*(X4) [21] primers and gel electrophoresis analysis were performed using the optimized primer sequences (Table S2). All experiments were performed with biological replicates.

### 2.2. Killing Kinetics Determination

The donor bacteria *E. coli* LD67-1, *E. coli* L65, *E. coli* RF2-1, and recipient bacteria *E. coli* EC600 were cultured to exponential phase and then cultured with different concentrations of AZT ranging from 0 to 1 *μ*g/mL. The corresponding CFUs per mL of the bacteria were counted by plate counting method at 0, 6, and 12 hr. All experiments were performed with three biological replicates.

### 2.3. Chemical Transformation Assay

The transformation system was established using competent cell *E. coli* DH5*α* as the recipient bacteria and plasmids pUC19, pBAD, and pWM91 as the DNA vectors. The plasmid was mixed with *E. coli* DH5*α* to reach the final concentration at 1 ng/*μ*L in the precooled tube. Then, different concentrations of AZT were added to the transformation system and incubated on ice for 30 min. After that, a 90s heat-shock with 42°C was conducted, followed by immediate placement on ice. After 5 min, 900 *μ*L LB broth was appended to the transformation system before being incubated at 37°C for 1 hr. The liquid was serially diluted and plated on the ampicillin-containing plates to select the transformants, and the recipient bacteria was determined on the LB agar plates, which contained no antibiotics. The transformation frequency was calculated through dividing the transformants by the recipient bacteria.

### 2.4. Measurement of Bacterial Respiration Levels

Bacterial respiration levels were determined using resazurin dye. *E. coli* RF2-1 and EC600 were washed with PBS to achieve OD_600_ of 0.5 and mixed with 10 *μ*L different concentrations of AZT in a 96-well plate with a dark bottom. Then, 0.1 *μ*g/mL resazurin dye was added to the mixed system. A 15 min continuous determination was conducted with an excitation wavelength of 550 nm and an emission wavelength of 590 nm.

Bacterial NADH was detected using the Detection Assay Kit according to the manufacturer's instructions. The donor and recipient bacteria were treated as described above. After amplification, centrifugation, and resuspending with PBS, bacteria were coincubated with different concentrations of AZT for 6 hr before centrifugation, and the supernatant was removed, respectively. Then, the lysis solution was added and centrifuged to collect the supernatant to determine NADH after heating it at 60°C. The intracellular NADH levels were measured when the absorbance was at 450 nm. Experiments were performed with three biological replicates.

### 2.5. Detection of Membrane Potential (*ΔΨ*) and *Δ*pH

*E. coli* RF2-1 and EC600 bacteria were incubated as described above to achieve OD_600_ of 0.5 after washing with PBS. 0.5 *μ*M DiSC_3_(5) (Aladdin, Shanghai, China) fluorescence dye was added and then cultured in the darkness for 30 min. After that, 190 *μ*L bacteria cells were mixed with 10 *μ*L AZT in a 96-well black plate. After incubating for 1 hr in the darkness, the fluorescence intensity was measured with the excitation wavelength of 622 nm and emission wavelength of 670 nm. All experiments were performed with three biological replicates.

For the detection of *Δ*pH, BCECF-AM (pH) fluorescence dye (Beyotime, Shanghai, China) was added to achieve the final concentration of 10 *μ*M for 30 min in the darkness. Intracellular changes in pH after the addition of different concentrations of AZT were monitored continuously for 10 min. Fluorescence intensity was measured by a Microplate reader (Tecan) with an excitation wavelength of 488 nm and emission wavelength of 535 nm.

### 2.6. RNA Extraction and RT-qPCR Determination

*E. coli* RF2-1 and EC600 were cultivated to exponential phase respectively, and resuspended to OD_600_ of 0.5 with PBS. Then 0, 0.0625, 0.25, and 1 *μ*g/mL AZT were added to the conjugation system. After incubation for 6 hr, the total RNA was extracted using the Total RNA Extraction Reagent (Vazyme) and quantified by the ratio of absorbance (260/280 nm) using a Nanodrop spectrophotometer (Thermo Scientific). Following the manufacturer's instruction, reverse transcription of 1 *μ*g RNA was performed using the HiScript® III RT SuperMix for qPCR (+gDNA wiper) Kits (Vazyme). RT-qPCR assay was performed by 7500 Fast Real-Time PCR System (Applied Biosystem, CA, USA) using the ChamQ™ Universal SYBR® Color qPCR Master Mix Kits (Vazyme) with the optimized primers. The relative expression levels of the target genes were normalized using the internal control gene (*16S rRNA*). The reaction conditions were set as follows: pre-denaturation step at 95°C for 30 s, and 40 cycles of denaturation for 10 s at 95°C, primer annealing for 30 s at 60°C, and extension for 30 s at 72°C. The primer sequences used in this study were shown in Table S3.

### 2.7. Swimming Motility Assay

LB broth with 0.3% agar powder was used to assess the swimming motility of *E. coli* RF2-1 and EC600. The agar plates were appended with 0, 0.0625, 0.25, and 1 *μ*g/mL AZT, respectively, and inoculated with 2 *μ*L bacteria cells stuck to the center of agar plates. After incubating at 37°C for 48 hr, the inhibition zones in the plates were filmed, and the bacteria zones were measured.

### 2.8. The Determination of ATP Level

Intracellular ATP levels of *E. coli* RF2-1 and EC600 bacteria were determined by the Enhanced ATP Assay Kits (Beyotime, Shanghai, China). The bacteria were cultured to stationary phase and then washed by PBS to achieve OD_600_ = 0.5. After being treated with 0, 0.0625, and 0.25 *μ*g/mL AZT for 1.5 hr, the bacteria were centrifuged, and the supernatants were removed. Then, the ATP lysis solution was added to release the ATP before centrifugation to collect the supernatant for the following step. Finally, 100 *μ*L ATP assay solution and 20 *μ*L lysed sample were added to the well according to the instruction book. The intracellular ATP levels were measured using a Microplate Reader (Tecan).

### 2.9. Metal Ions Measurement

Ten millimolar metal ions, including Na^+^, K^+^, Mg^2+^, and Ca^2+^, were added to the conjugation system after mixing the *E. coli* RF2-1 and EC600 bacteria. The process of conjugation assay was in accordance with Section 2.1, except for the conjugation media, which was LB broth instead of PBS. All experiments were performed with biological replicates.

### 2.10. Bacterial Passaging Assay and SNP Analysis

Sequential passaging culture of *E. coli* RF2-1 and *E. coli* RS3-1 was conducted in the presence of subinhibitory concentrations of AZT. The serial bacteria passaging was repeated every 12 hr, and the MIC was determined by broth microdilution every 24 hr. Then, the conjugative transfer frequency between the original and passaged bacteria was compared. In addition, we extracted the original and passaged bacterial DNA to conduct Illumina sequencing and SNP analysis to explore the distinction of different bacteria.

### 2.11. Docking Analysis

A template was created using the crystal structure of the thymidine kinase. Then, thymidine kinase and AZT were molecularly docked using the Autodock Vina tool without the use of water molecules. In a 2D graphic, Discovery Studio 4.5 depicted the interactions of AZT with the residues of the binding sites in thymidine kinase.

### 2.12. *In Vivo* Assay

Female ICR mice (*n* = 9 per group) were intraperitoneally infected with a single dose of 2.0 × 10^8^ CFUs/mL bacteria suspension of the donor (*E. coli* LD67-1, *E. coli* L65, *E. coli* RF2-1) and recipient (*E. coli* EC600) bacteria mixture (1 : 1). At 15 min post infection, a dose of 200 *μ*L PBS or AZT (10 mg/kg) was injected intraperitoneally, respectively. After 24 hr, mice were euthanized by cervical dislocation. The liver was aseptically removed, homogenized, serially diluted, and plated on the antibiotic-containing agar for CFUs titers of conjugants as described in our previous study.

### 2.13. Statistical Analysis

Statistical analysis was performed using GraphPad version 9.0 software. All data were presented as mean ± SD. Unpaired *t*-test (normally distributed data) between two groups and one-way ANOVA or two-way ANOVA analysis among multiple groups were used to calculate *P*-values. Differences with *P*  < 0.05 were considered significant. Significance levels were indicated by numbers of asterisks:  ^*∗*^*P* < 0.05,  ^*∗∗*^*P* < 0.01,  ^*∗∗∗*^*P* < 0.001, and  ^*∗∗∗∗*^*P* < 0.0001. n.s. not significant.

## 3. Results

### 3.1. AZT Prevents the Horizontal Transfer of ARGs

**Figure 1:**
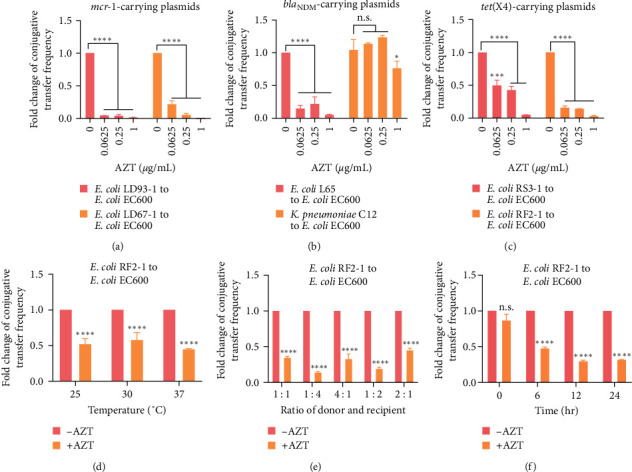
Effects of AZT on the conjugative transfer of different types of plasmids carrying multiple antibiotic resistance genes. (a–c) Fold changes of conjugative transfer frequency of *mcr-1*-bearing IncX4 and IncI2 plasmids (a), *bla*_NDM−5_-bearing IncX plasmids (b), and *tet*(X4)-bearing IncFI and IncFII plasmids (c) from different bacteria species to *E. coli* EC600. (d–f) Effects of 0.25 *μ*g/mL AZT on conjugative transfer of *tet*(X4)-positive plasmid from *E. coli* RF2-1 to *E. coli* EC600 under different conditions, including mating temperature (d), bacteria ratio of donor and recipient (e), and mating time (f). Significant differences were evaluated by two-way ANOVA analysis and shown with  ^*∗*^*P* < 0.05,  ^*∗∗∗*^*P* < 0.001, and  ^*∗∗∗∗*^*P* < 0.0001. n.s. not significant.

Prior to conjugative transfer assays, we conducted the time-killing kinetics to determine whether the tested concentrations of AZT influence bacterial growth. The results indicated that adding selected concentrations of AZT had no effect on bacterial growth (Figure S1). Next, we investigated the impact of AZT on the conjugative transfer of plasmids carrying clinically important ARGs, including *mcr-1*, *bla*_NDM−5_, and *tet*(X4) genes, which confer bacterial resistance to colistin, meropenem, and tigecycline, respectively. Intriguingly, we found that AZT caused a dose-dependent reduction of the conjugative transfer frequency in two *mcr-1* carrying plasmids (belong to IncX4 and IncI2 types) and two *tet*(X4)-bearing plasmids (IncF type) (Figures 1(a), 1(c), S2(a), and S2(c)). With regard to *bla*_NDM−5_-positive plasmids, AZT inhibited the conjugal transfer of *bla*_NDM−5_-bearing IncX3 plasmid of *E. coli* L65. However, only 1 *μ*g/mL AZT restrained the plasmid transfer from *K. pneumoniae* C12 to *E. coli* EC600 (Figures 1(b) and S2(b)). Meanwhile, MIC determination and PCR analysis were conducted to confirm the accuracy of transconjugants. MIC results showed that these transconjugants displayed corresponding resistance phenotypes to colistin, meropenem, tigecycline, or rifampicin, respectively (Figure S3(a)). Consistently, the corresponding ARGs were confirmed from the agarose gel electrophoresis results (Figure S3(b)).

Although the inhibition effect of AZT is effective for the clinical strains, whether it is due to the genetic background of the clinical strains is still unknown. Based on this, we applied the engineered bacteria *E. coli* TOP10 carrying three different clinical plasmids as the donor to exclude the impact of host bacteria. Similarly, we found that AZT could also exhibit a dose-dependent inhibitory effect on conjugative transfer (Figure S4).

Furthermore, we assessed the inhibitory effect of AZT under varying mating conditions, including mating temperature, bacteria ratio of donor and recipient cells, and mating time, providing a perspective for the optimal conditions for the potential application of AZT in clinical settings. Specifically, AZT inhibited the plasmid transfer frequency obviously from *E. coli* RF2-1 to *E. coli* EC600 at different mating temperatures (from 25 to 37°C), and 37°C was often regarded as the optimum temperature (Figures 1(d) and S2(d)). Also, the inhibitory effect of AZT on conjugation was observed in five different ratios of donor and recipient cells (Figures 1(e) and S2(e)). The mating time also played an important role in the conjugation as the effects of AZT were strengthened along with prolonged mating time (Figures 1(f) and S2(f)).

**Figure 2:**
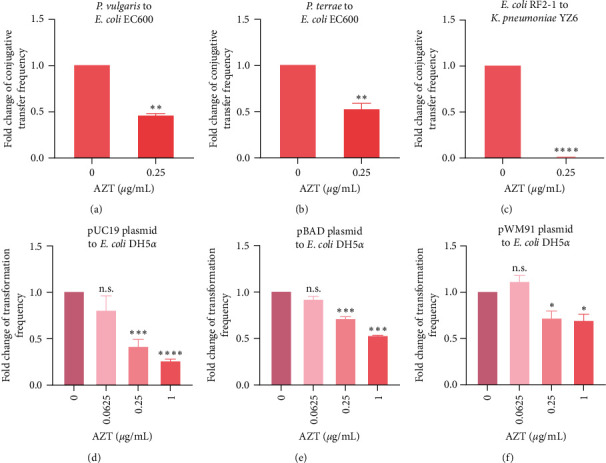
AZT represses horizontal gene transfer through conjugation and chemical transformation. (a and b) Fold changes of conjugative transfer frequency of resistance plasmids from *P. vulgaris* (a) and *P. terrae* (b) to *E. coli* EC600 under 0.25 *μ*g/mL AZT. (c) Fold changes of conjugative transfer frequency of *tet*(X4)-positive plasmid from *E. coli* RF2-1 to *K. pneumoniae* YZ6 following treatment with AZT. (d–f) Fold changes of transformation frequency from pUC19 (d), pBAD (e), and pWM91 (f) plasmids to *E. coli* DH5*α* with the addition of AZT. Unpaired *t*-test between two groups or one-way ANOVA among multiple groups were used to calculate *P*-values ( ^*∗*^*P* < 0.05,  ^*∗∗*^*P* < 0.01,  ^*∗∗∗*^*P* < 0.001, and  ^*∗∗∗∗*^*P* < 0.0001. n.s. not significant).

Next, we evaluated ARGs transfer frequency exposed to AZT under different conditions. We first determined the intergenera conjugative transfer frequency from two kinds of *tet*(X4)-positive *Proteus*, including *P. vulgaris* and *P. terrae*, to *E. coli* EC600. As expected, a low concentration of AZT (0.25 *μ*g/mL) significantly repressed the transfer of *tet*(X4)-carrying plasmids from *Proteus* to *E. coli* (Figures 2(a), 2(b), S5(a), and S5(b)). In addition, AZT also restrained the plasmid transfer from *E. coli* RF2-1 to *K. pneumoniae* YZ6 by 10.0-folds (Figures 2(c) and S5(c)). AZT also inhibited the transfer of three engineered plasmids carrying *amp* resistance gene, including pUC19, pBAD, and pWM91, through chemical transformation (Figures 2(d), 2(e), 2(f), S5(d), S5(e), and S5(f)). Taken together, these results demonstrated the broad-spectrum and excellent inhibitory effect of AZT on the horizontal transmission of ARGs.

### 3.2. AZT Prevents Conjugative Transfer by Dissipating Bacterial PMF

**Figure 3:**
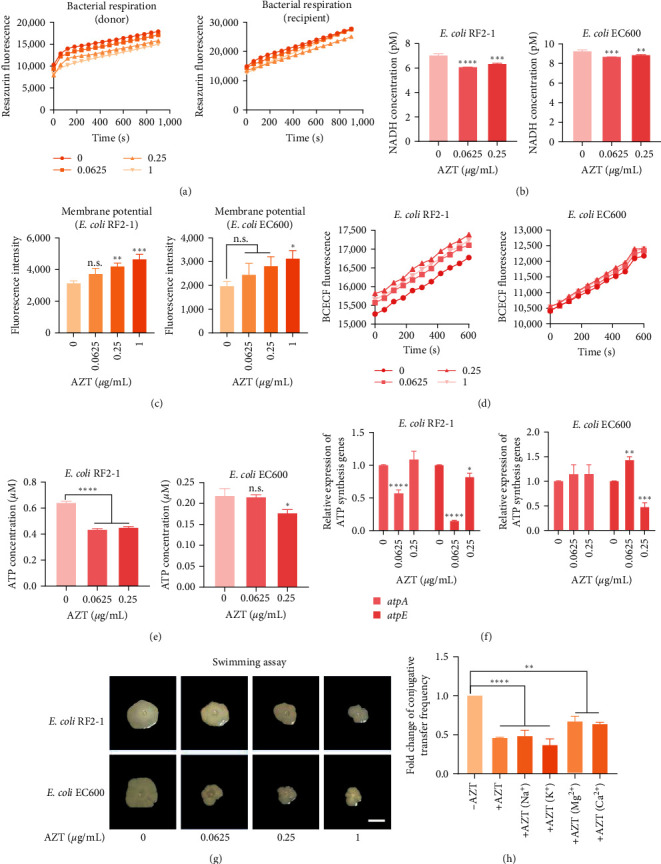
AZT prevents conjugative transfer via disrupting bacterial proton motive force. (a) The bacterial respiration level of donor and recipient bacteria, along with different concentrations of AZT (0–1 *μ*g/mL), was determined by monitoring the reduction of resazurin to resorufin. (b) NADH concentrations in *E. coli* RF2-1 and *E. coli* EC600 when exposed to AZT. (c) Membrane potential changes of *E. coli* RF2-1 and *E. coli* EC600 after treatment with AZT, detected using DiSC_3_ (5) fluorescence dye. (d) The intracellular pH changes in the two bacteria were determined using BCECF fluorescence dye. (e) The ATP level of *E. coli* RF2-1 and *E. coli* EC600 after exposure to different concentrations of AZT. (f) Relative expression of ATP synthesis-related genes, determined using RT-qPCR. (g) Swimming motility assay after exposure to increasing concentrations of AZT. The plates were prepared using 0.3% agar and inoculated with 2 *μ*L bacteria suspensions. Scar bar, 1 cm. (h) Fold changes of conjugative transfer frequency with AZT when appended with monovalent and divalent ions (10 mM). Significant differences were evaluated by one-way ANOVA or two-way ANOVA analysis and shown with  ^*∗*^*P* < 0.05,  ^*∗∗*^*P* < 0.01,  ^*∗∗∗*^*P* < 0.001, and  ^*∗∗∗∗*^*P* < 0.0001. n.s. not significant.

Bacterial cells largely depend on multiple cellular events to maintain normal physiological activities, including bacterial electric transfer chain (ETC), PMF, and ATPase activities [22]. ETC, also called the respiratory chain, has high-productivity efficiency and is the main way for bacteria to obtain energy [23], which is important for the conjugation process. Therefore, we first detected the bacterial respiration levels using resazurin dye. As expected, we found a decrease in the resazurin fluorescence along with 0–1 *μ*g/mL AZT addition, indicating a reduction of bacterial respiration level (Figure 3(a)). Moreover, during aerobic respiration, NADH (nicotinamide adenine dinucleotide), mainly involved in material and energy metabolism in bacterial cells and produced in the citric acid cycle, would transform to NAD^+^, its oxidative state [24, 25]. As shown in Figure 3(b), AZT repressed the production of NADH with the addition of AZT, suggesting the suppression of the tricarboxylic acid (TCA) cycle and the disruption of ETC activities.

Meanwhile, protons would be pumped out of the cytoplasmic membrane to form a transmembrane electrochemical gradient through ETC, named PMF. As one of the critical components of PMF, the electric potential (*Δψ*) could be detected with a fluorescent dye DiSC_3_(5) [26]. As shown in Figure 3(c), we found that the fluorescence intensity of the *E. coli* RF2-1 was escalated along with AZT addition, indicating the dissipation of *Δψ*. However, only 1 *μ*g/mL AZT upregulated the fluorescence intensity of *E. coli* EC600. To maintain the stability of PMF, the dissipation of *Δψ* would be compensated by an increase in transmembrane proton gradient (*Δ*pH). Consistently, we detected the *Δ*pH across the cell membrane using a BCECF fluorescence probe and found a promotion effect in donor bacteria, suggesting the increased *Δ*pH. Also, this phenomenon was not found in recipient bacteria (Figure 3(d)).

PMF is also regarded as a necessary driving force of intracellular ATP synthesis and bacterial motility [27, 28]. To further evaluate the downstream processes caused by AZT-induced PMF dissipation, we first performed a swimming assay to detect bacterial flagellar motility. When exposed to different concentrations of AZT, the swimming zones were repressed obviously in donor and recipient bacteria (Figure 3(g)). Thereafter, the determination of intracellular ATP levels suggested that the production of ATP was decreased in donor and recipient bacteria. As shown in Figure 3(e), the ATP level was downregulated in the bacteria, and the relative expression of *atpA* and *atpE* genes was also inhibited (Figure 3(f)). These results indicated that the dissipated PMF induced by AZT finally limited the energy supply. To further verify the role of dissipated PMF in the inhibitory effect of AZT on conjugation, we tried to recover the dissipated PMF by adding exogenous ions. Consistently, the supplementation of metal ions, especially divalent ions (Mg^2+^ and Ca^2+^), could remit the suppression of AZT on transmission to a large extent (Figure 3(h)), indicating the critical role of stable PMF in the conjugation process.

### 3.3. AZT Suppresses Bacterial Secretory System

**Figure 4:**
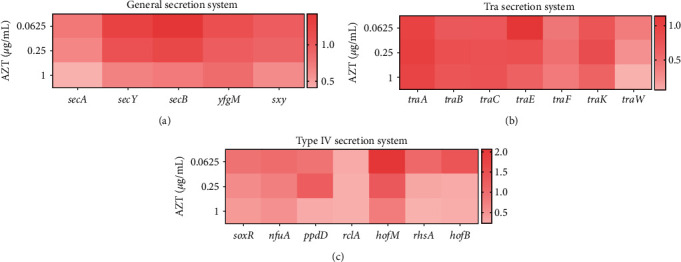
RT-qPCR analysis of the secretion system-related gene expression in *E. coli* RF2-1 under AZT treatment. (a–c) Fold changes of the relative mRNA expression of general secretion system (a), Tra secretion system (b), and type IV secretion system (c) related genes in *E. coli* RF2-1 after treatment with AZT.

Bacterial pathogens utilize several methods to invade mammalian hosts and damage tissue cells. Bacterial proteins across phospholipid membranes, namely bacterial secretion systems, have been recognized as an essential component of this process [29]. Mounting evidence suggests that bacteria absorb the substances through a secretion system, which is majorly dependent on the cell membrane channels [30]. Among the secretion systems, the Type IV secretion system (T4SS), as a transmembrane channel structure, could deliver substrates containing DNA, protein, and other macromolecules to the target cells [31], and the general secretion system participates in the formation of T4SS [4, 32]. In *E. coli*, the general secretory system exports proteins from the cytoplasm before they are stably folded [33]. Considering the indispensable role of the secretion system in conjugation, we detected the relative expression of the secretion system-related genes, including the Tra and T4SS secretion system. Specifically, *secA*, which is an inner membrane component of the Sec protein secretion system and is peripherally linked with the multi-subunit translocation apparatus SecYEG [34, 35], was inhibited obviously along with AZT. Genes related to the positive regulation of the general secretion, such as *secY* and *sxy*, were also downregulated, indicating the inhibition of HGT (Figure 4(a)). In addition, we determined the Tra system-related genes, such as the *traC*, *traW*, and *traF* gene, which also exhibited an evident downregulation in the IncF plasmid (Figure 4(b)). The expression of the *ppdD* gene, related to the type IV pilus [36], was also downregulated along with AZT. The *soxR* gene, contributing to the formation of DNA, was suppressed obviously in *E. coli* RF2-1. Some other genes, including *rhsA* and *hofB* gene, also showed an inhibition phenomenon with the addition of AZT (Figure 4(c)). Taken together, these results denoted that the downregulated expression of bacteria secretory system may be one of the reasons why AZT suppressed the transmission of ARGs.

### 3.4. AZT Inhibits Conjugation by Acting on Bacterial Thymidine Kinase

**Figure 5:**
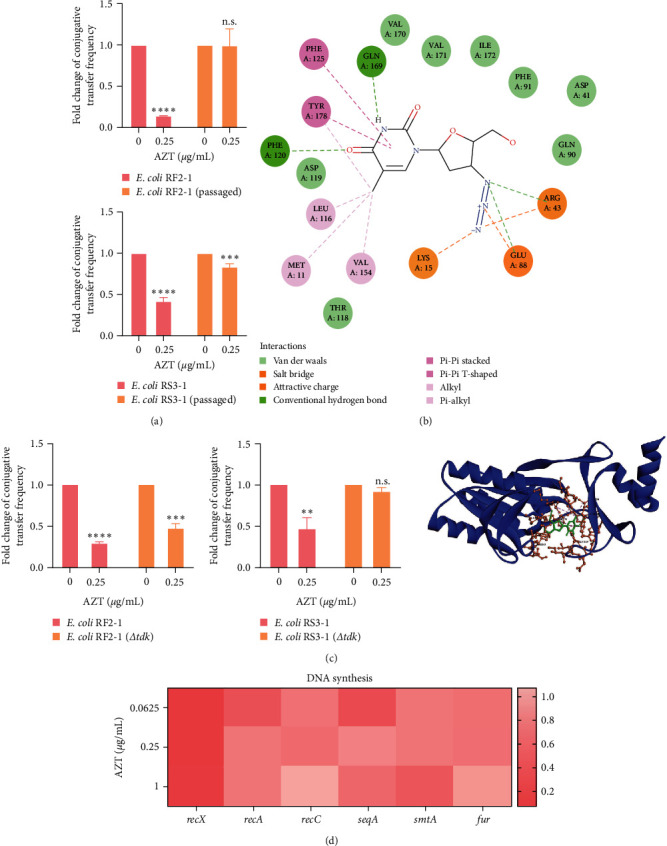
AZT inhibits conjugative transfer by targeting thymidine kinase. (a) Fold changes of conjugative transfer frequency in original and passaged bacteria when exposed to 0.25 *μ*g/mL AZT. (b) Silico docking analysis between thymidine kinase and AZT, with a binding energy of −9.2 kcal/mol. (c) The effect of *tdk* gene deletion on the inhibitory effect of AZT on conjugation. (d) Relative expression of DNA synthesis-related genes of *E. coli* RF2-1 treated by AZT. Significant differences were evaluated by two-way ANOVA analysis and shown with  ^*∗∗*^*P* < 0.01,  ^*∗∗∗*^*P* < 0.001, and  ^*∗∗∗∗*^*P* < 0.0001. n.s. not significant.

Previous assays indicated that the resistance against AZT under its own pressure could be generated rapidly [37]. Therefore, we conducted the sequential culturing of *E. coli* RF2-1 and *E. coli* RS3-1 in the presence of subinhibitory concentrations of AZT. The serial passaging of bacteria was repeated every 12 hr, and the MIC value was determined by broth microdilution daily. As a result, AZT-resistant *E. coli* cells evolved along with continuous passages under increasing sub-MIC concentrations of this drug. As shown in Figure S6, two clinical bacteria, *E. coli* RF2-1 and *E. coli* RS3-1, obtained AZT resistance higher than 2,500 folds as the initial MIC was no more than 10 *μ*g/mL within 20 passages. Furthermore, we used these two passaged bacteria as donor bacteria and *E. coli* EC600 as recipient bacteria to conduct conjugation. Strikingly, the inhibitory effect of AZT on the gene transfer was apparently declined (Figure 5(a)). Next, we conducted SNP analysis to explore the mutation site and locked the thymidine kinase gene *tdk* as the primary site (Table 1). Thymidine kinase plays a central role in DNA synthesis during cell division and is involved in the mediation of deoxythymidine [38]. In order to better understand how AZT inhibits the enzymatic activity of thymidine kinase, we conducted *in silico* docking analysis with thymidine kinase as receptor and AZT as the ligand, showing that AZT had a high affinity for thymidine kinase with a binding energy of −9.2 kcal/mol. Specifically, AZT could attach to thymidine kinase through Van del Waals (ASP119, VAL170, ILE172, PHE91, ASP41, and GLN90), hydrogen bond with PHE120 and GLN169, and attractive charge with LYS15, GLU88, and ARG43 (Figure 5(b)).

In addition, we constructed the *Δtdk* strain carrying *tet*(X) plasmid as the donor bacteria and performed the conjugation transfer experiments. Consequently, we found that the inhibitory effect of AZT was drastically weakened in gene deletion bacteria, indicating the importance of the interaction of AZT-thymidine kinase in the decreased transmission of AZT (Figure 5(c)). Due to the importance of *tdk* gene in DNA synthesis, we also determined the expression of related genes. As expected, AZT inhibited the Rec system and the expression of *seqA*, *smtA*, and *fur*-related genes in donor bacteria (Figure 5(d)). These results suggested that AZT inhibited conjugation by acting on bacterial thymidine kinase.

### 3.5. AZT Blocks Conjugative Transfer of ARGs *In Vivo*

**Figure 6:**
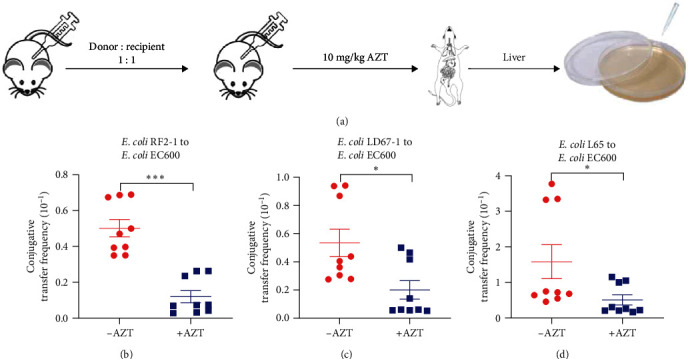
AZT prevents the conjugative transfer of resistance plasmids *in vivo*. (a) Protocols of AZT-treated administration in mice (*n* = 9 biologically independent animals per group). Prior to AZT treatment, mice were supplied with a single intraperitoneal (*i.p*.) administration of donor: recipient (1 : 1). (b–d) Conjugative transfer frequency of resistance plasmids from three donor bacteria, including *E. coli* RF2-1 (b), *E. coli* LD67-1 (c), and *E. coli* L65 (d), to the recipient bacteria *E. coli* EC600, respectively, in mice livers. *P* values were determined using the Mann–Whitney *U*-test.

The results mentioned above confirmed that AZT suppressed the conjugation frequency of multiple ARGs within and across genera *in vitro*. The prevalent clinical application of AZT, however, highlights the necessity to identify its effect *in vivo* [39]. In this work, three types of clinical strains were selected as donor bacteria, with the *E. coli* EC600 as recipient bacteria. After adaptive culture for one week, the mice were intra-abdominal infected with the donor and recipient bacteria mixture (1 : 1). Next, a single dose of AZT (10 mg/kg) was intraperitoneally injected at 15 min post-infection, and the equivalent PBS was set as a vehicle. The number of colonies in the livers was determined using specific antibiotics-containing plates, and the corresponding conjugative transfer frequency was calculated (Figure 6(a)). As shown in Figure 6(b), AZT treatment (10 mg/kg) remarkably reduced the conjugative transfer frequency of *tet*(X4) carrying plasmid by more than five-fold. With regard to *mcr-1* or *bla*_NDM−5_-harboring plasmids, only a two-fold reduction of conjugal frequency exposure to AZT was observed (Figures 6(c) and 6(d)). Taken together, these results verified the capacity of AZT to inhibit the conjugation process of clinical strains *in vivo*.

## 4. Discussion

Accumulating evidence suggests that the overuse and misuse of antibiotics have been deteriorating the situation by contributing to the dissemination of ARGs, which pose a constant threat to global public health [40, 41]. In addition to antibiotics, nonantibiotic compounds have the potential to expedite the prevalence of ARGs. For example, preservatives, including sodium nitrite, sodium benzoate, and triclocarbon, were found to enhance the spread of extracellular ARGs through conjugation via different mechanisms [42]. Acetaminophen, an antipyretic agent available in numerous prescriptions, promoted the horizontal transfer of plasmid-borne multiple ARGs [43]. Despite the fact that the promotive effect of several substrates on conjugation has been confirmed, only a few compounds have been identified as HGT inhibitors. As a nucleoside reverse transcriptase inhibitor, AZT is involved in HIV-1 therapy and is combined with fosfomycin against MDR *Enterobacterales* [44, 45], whereas its potential in preventing plasmid transmission remains poorly understood.

In this study, we revealed the capability of AZT to block the horizontal transfer of ARGs between intra- and interspecies. Notably, the suppression effect of AZT could be found in various clinically relevant plasmids, including *mcr-1*-bearing IncX4 and IncI2 plasmids, *bla*_NDM−5_-bearing IncX plasmids, and *tet*(X4)-positive plasmids. Also, AZT inhibited the transmission across genera from *E. coli* to *K. pneumoniae* and *Proteus* to *E. coli*. In agreement with previous studies, the conjugation frequency across genera was inferior to the within genera [12, 41], possibly due to reproductive isolation and species barriers. In addition to conjugation, we also demonstrated that AZT could prevent the spread of resistance plasmids through chemical transformation, in which bacteria take up free DNA from the environment and recombine it into their genome [46]. However, the effect of AZT on natural transformation remains to be evaluated in the future study. Considering that HGT could take place in various conditions due to the ubiquitous resistance, we also confirmed that the inhibitory effect of AZT could be detected in diverse environmental conditions, thereby providing an alternative approach to effectively limit and control the prevalence of ARGs. Specifically, the action of AZT was more obvious when the conjugation temperature was at 37°C, which was more suitable for bacterial growth in mammals and environments. In a murine model, we demonstrated that a single dose of AZT at 10 mg/kg, which was comparable to the clinical dosage of AZT to treat HIV infection, effectively prevented the transfer of three resistance plasmids.

Mechanistic studies revealed that AZT disrupted bacterial TCA, decreased the NADH required for respiratory chain activities, and dissipated PMF through collapsing *ΔΨ*. These findings also indicated the importance of energy metabolism for bacterial conjugation. Consistently, our previous study also revealed that accelerated bacterial energetic metabolism contributes to the horizontal transfer of ARGs [47]. Most importantly, the dissipation of bacterial PMF, a proton electrochemical gradient across the cell membrane, was responsible for the inhibitory effect of AZT on conjugation. Consistent with our findings, a previous study screened and identified potent inhibitors of *S. pneumoniae* competence, which regulated the transformation machinery and prevented gene transfer by inhibiting bacterial PMF [48]. These evidences revealed that PMF could serve as a critical target for the identification of HGT inhibitors [49].

Besides, the bacterial conjugative transfer system is a member of the secretion system, which plays a critical role in the spread of antibiotic resistance and virulence [50, 51]. One distinctive feature of the secretion system is that it is a multifunctional transmembrane channel consisting of numerous components, which enables it to translocate DNA, proteins, and other macromolecules [52, 53]. Considerable attention has been drawn to the conjugation system of the secretion system for its ability to disseminate mobile genetic materials, many of which accelerate resistance transmission [52]. For example, triclosan enhanced the spread of extracellular ARGs through upregulating the expression of secretion system-related genes [54]. Our research verified that the inhibitory effect of AZT on conjugation was associated with the downregulation of the bacterial secretion system, which functions through an envelope-spanning multiprotein channel like a pilus structure to contact with the recipient cell surface [55].

Most importantly, our results revealed that the mutation and deletion of the thymidine kinase gene conferred bacterial resistance to AZT and substantially declined its inhibition on conjugation, suggesting that thymidine kinase may be the potential target of AZT. It is plausible that the small molecule AZT could bind to the thymidine kinase and disrupt the synthesis of bacterial DNA, thereby inhibiting the horizontal transfer of resistance plasmids. Additionally, it is noteworthy that the rapid development of AZT resistance under continuous drug stimulation may limit the long-term usage of AZT in clinical practice. Therefore, more animal model experiments and *in vivo* evaluation of AZT are required to provide comprehensive clinical guidance for its future applications.

In conclusion, our study demonstrates that the anti-HIV drug AZT can serve as a broad HGT inhibitor, which effectively inhibits the transmission of ARGs both in intrageneric and intergeneric by disrupting bacterial PMF, restraining the secretion system, and targeting thymidine kinase, thus providing a promising strategy to tackle AMR.

## Figures and Tables

**Table 1:** SNP analysis between the original and AZT-passaged bacteria.

Strains/genes	Products	Nucleotide change	POS	Description
*E. coli* RS3-1				
* vgrG*	VgrG protein	A⟶G	118/642	Type VI secretion system tip protein
* Tdk*	Thymidine kinase	A⟶C	205,578	DNA synthesis
*E. coli* RF2-1				
* vgrG*	VgrG protein	C⟶T	859	Type VI secretion system tip protein
* tolA*	TolA protein	C⟶T	1,033	Inner membrane protein
* yjjG*	Pyrimidine 5′-nucleotidase YjjG	CT⟶C	38,711	DNA double helix structure
* yqiJ*	Inner membrane protein YqiJ	T⟶C	1,081	Inner membrane protein
* tdk*	Thymidine kinase	C⟶T	201,788	DNA synthesis

## Data Availability

The data used to support the findings of this study are included within the article and supplementary information.

## Supplementary Materials

Supplementary Materials Table S1: the information of strains and plasmids used in this study. Tables S2 and S3: primer sequences used in this study. Figure S1: killing kinetics of donor and recipient bacteria after exposure to different concentrations of AZT. Figure S2: effects of AZT on the conjugation of different types of plasmid-borne ARGs. Figure S3: MIC determination and PCR verification of donor, recipient and transconjugants bacteria under different concentrations of AZT. Figure S4: fold changes of conjugative transfer frequency of the engineered bacteria *E. coli* TOP10 carrying clinical tet(X4) plasmids after exposure to AZT. Figure S5: inhibitory effects of AZT on conjugation and chemical transformation. Figure S6: Resistance development of *E. coli* to AZT after 10 days of serial passaging.
