# Supplementary material for: A Broad-Spectrum Horizontal Transfer Inhibitor Prevents Transmission of Plasmids Carrying Multiple Antibiotic Resistance Genes
Source: Transbound Emerg Dis. 2024 May 30;2024:7063673. doi: 10.1155/2024/7063673 (PMC12017466; doi:10.1155/2024/7063673)
Supplement: Supplementary Materials — Table S1: the information of strains and plasmids used in this study. Tables S2 and S3: primer sequences used in this study. Figure S1: killing kinetics of donor and recipient bacteria after exposure to different concentrations of AZT. Figure S2: effects of AZT on the conjugation of different types of plasmid-borne ARGs. Figure S3: MIC determination and PCR verification of donor, recipient and transconjugants bacteria under different concentrations of AZT. Figure S4: fold changes of conjugative transfer frequency of the engineered bacteria E. coli TOP10 carrying clinical tet(X4) plasmids after exposure to AZT. Figure S5: inhibitory effects of AZT on conjugation and chemical transformation. Figure S6: Resistance development of E. coli to AZT after 10 days of serial passaging. [file 7063673.f1.docx]

**Supplementary Materials**

**Table S1.** **The information of strains and plasmids used in this study.**

| Strains/plasmids | Genotypes | Sources | Resistance genes |
| --- | --- | --- | --- |
| *E. coli* LD93–1 | IncX4 | Pere David's Deer | *mcr-1* |
| *E. coli* LD67–1 | IncI2 | Pere David's Deer | *mcr-1* |
| *E. coli* L65 | IncX3 | Goose farm | *bla*_NDM-5_ |
| *K. pneumoniae* C12 | IncX4 | Goose farm | *bla*_NDM-5_ |
| *E. coli* RS3–1 | IncFI | Slaughter house | *tet*(X4) |
| *E. coli* RF2–1 | IncFII | Goose farm | *tet*(X4) |
| *K. pneumoniae* YZ6 | pKPHS | Human source | *hyg* |
| *Proteus* F70-4 | – | Slaughter house | *tet*(X4) |
| *Proteus* IC17-4 | – | Slaughter house | *tet*(X4) |
| *E. coli* EC600 | – | Engineering bacteria | – |
| *E. coli* DH5α | – | Engineering bacteria | – |
| pUC19 plasmid | – | Engineering plasmid | *amp* |
| pBAD plasmid | – | Engineering plasmid | *amp* |
| pWM91 plasmid | – | Engineering plasmid | *amp* |
| *E. coli* TOP10 | – | This study | – |

**Table S2. Primer sequences used in this study for PCR verification.**

| Genes | Sequences | Product (bp) | References |
| --- | --- | --- | --- |
| *mcr-1* | F: AGTCCGTTTGTTCTTGTGGC | 320 | ^1^ |
|  | R: AGATCCTTGGTCTCGGCTTG |  |  |
| *bla*_NDM-5_ | F: CACCTCATGTTTGAATTCGCC | 1000 | ^2^ |
|  | R: CTCTGTCACATCGAAATCGC |  |  |
| *tet*(X4) | F: TGAACCTGGTAAGAAGAAGTG | 610 | ^3^ |
|  | R: CAGACAATATCAAAGCATCCA |  |  |

1. Rebelo, A. R. *et al.* Multiplex PCR for detection of plasmid-mediated colistin resistance determinants, *mcr-1*, *mcr-2*, *mcr-3*, *mcr-4* and *mcr-5* for surveillance purposes. *Euro surveillance* **23**, 17-00672 (2018).

2. Liu, Z. *et al.* Emergence of IncX3 plasmid-harboring *bla*_NDM-5_ dominated by *Escherichia coli* ST48 in a goose farm in Jiangsu, China. *Front Microbiol* **10**, 2002 (2019).

3. Sun, J. *et al.* Plasmid-encoded *tet*(X) genes that confer high-level tigecycline resistance in *Escherichia coli*. *Nat Microbiol* **4**, 1457-1464 (2019).

**Table S3.** **Primer sequences used in this study for RT-qPCR analysis.**

| Genes | Sequences | Product (bp) |
| --- | --- | --- |
| *secA* | F: GCGTCTTTGGTATGCGTCAC | 109 |
|  | R: GTTGCGGTCAGGGTTTTTCC |  |
| *secY* | F: CGTTCTACTTCGGTGGGACC | 131 |
|  | R: GTAGCCTTTCAGGTTCGCCT |  |
| *secB* | F: AGTTCAGCAGGGCGGTATTT | 106 |
|  | R: CTCACGAGCATACGGGAACA |  |
| *yfgM* | F: ACTGGCTGTTGGGGTGATTT | 100 |
|  | R: ATAGGCAAGAGAAGCGGAGC |  |
| *sxy* | F: ACGGTGTTTGCGATGGTTTC | 122 |
|  | R: GTAACGGATCGGCCACACTT |  |
| *atpE* | F: ACGCCAACCATTTCTGACGA | 141 |
|  | R: AGACCCAGACCTACAGCGAT |  |
| *atpA* | F: GTGGTATCCGTACCGCTCTG | 73 |
|  | R: TCGTCAAGGTCGGATGCAAA |  |
| *soxR* | F: ATCCGTAACAGCGGCAATCA | 148 |
|  | R: TCGCACTTAACGTATGCCCT |  |
| *nfuA* | F: CCGAACGCCAAAATGCGTAA | 87 |
|  | R: CTGTGGGTTGATCTGCGACT |  |
| *ppdD* | F: ACCACCACCCGCTATGTTTC | 74 |
|  | R: TTGAGACTTTCTTGCCCGGT |  |
| *rclA* | F: CAATGTATGGCGGGACCTGT | 104 |
|  | R: TTTACGCTGTATGGCACGGA |  |
| *hofM* | F: ATGCTTTTTGCAACGCTGGT | 72 |
|  | R: GCATCAACAATCCGCCCATC |  |
| *rhsA* | F: TGCGCCGGGAATATGATGAA | 105 |
|  | R: CGCAGGGTAAGTCACTGTGT |  |
| *rhsB* | F: TCGCTATCTTTACGACCCGC | 147 |
|  | R: TTCTGTATCGTGGTCAGCCG |  |
| *hofB* | F: TAGTGCCCTGCAAAAGCTGA | 147 |
|  | R: CGCAATAACGCACGCAAAAC |  |
| *traA* | F: TGGCAAGTGGTAACTCCACG | 85 |
|  | R: GTACATGACAGCACCGACCA |  |
| *traC* | F: AACTCTGATGAGCCGTCCCT | 104 |
|  | R: AGGCTGAACATCACCGCAA |  |
| *traW* | F: ATCTGTGGCCGGTAAAGGAG | 81 |
|  | R: CCATCTCACCGGACTGCTC |  |
| *traF* | F: GTTCTTTTACCGGGGGCAGG | 111 |
|  | R: ATCACGCCATCCACGGAAA |  |
| *traB* | F: CCATTGTGAAACGCAAGCAGT | 82 |
|  | R: AGATACAGTGCCCCACCAATC |  |
| *traK* | F: AATGGTGAACGGTGGTCAGG | 71 |
|  | R: CACCGGGAACGGTAAACAGA |  |
| *traE* | F: GGCGCTGTCATTTATTGCCC | 77 |
|  | R: ATTGCAGAAGCGCCTGATGT |  |
| *recA* | F: GCCTCTGTTCGTCTCGACAT | 143 |
|  | R: TCGCCGTAGAGGATCTGGAA |  |
| *recX* | F: GCAAAGGTTATGGACCTGCG | 81 |
|  | R: GCATCGCTTTTTCTGTCGCT |  |
| *recC* | F: ACATCACCGAAGAAGGGCTG | 75 |
|  | R: CGTCATCTATGCCCCAACGA |  |
| *seqA* | F: TTGCCAGCCACACTAAGCAT | 114 |
|  | R: CAACGCGAACCTCTTTCGTC |  |
| *smtA* | F: ACAGGCTATTCTGTGGCAGG | 74 |
|  | R: CATCCAGCACACGCAGTTTT |  |
| *fur* | F: GAACCAGTTTGACGACGCTG | 111 |
|  | R: CAGTCGAGGCAGATCAGGTG |  |
| *16S rRNA* | F: CCTACGGGAGGCAGCAG | 194 |
|  | R: ATTACCGCGGCTGCTGG |  |





**Figure S1. Killing kinetics of** **donor and recipient bacteria after exposure to different concentrations of AZT.**

The CFUs/mL of donor **(A-C)** and recipient bacteria **(D)** were determined at 0, 6 and 12 h.





**Figure S2. Effects of AZT on the conjugation of different types of plasmid-borne ARGs.**

**(A)** Conjugative transfer frequency of *mcr-1*-bearing IncX4 and IncI2 plasmids in the presence of AZT. **(B)** Conjugative transfer frequency of *bla*_NDM-5_-bearing IncX plasmids from different bacteria species to *E. coli* EC600 in the presence of AZT. **(C)** Conjugative transfer frequency of *tet*(X4)-bearing IncFI and IncFII plasmids after exposure to AZT ranging from 0 to 1 μg/mL. **(D-F)** The effects of 0.25 μg/mL AZT on conjugative transfer frequency under different conditions including mating temperature **(D)**, ratio of donor and recipient bacteria **(E)**, and mating time **(F)**.

Significant differences were evaluated by two-way ANOVA analysis and shown with **P* < 0.05, ***P* < 0.01, ****P* < 0.001 and *****P* < 0.0001. n.s. not significant.


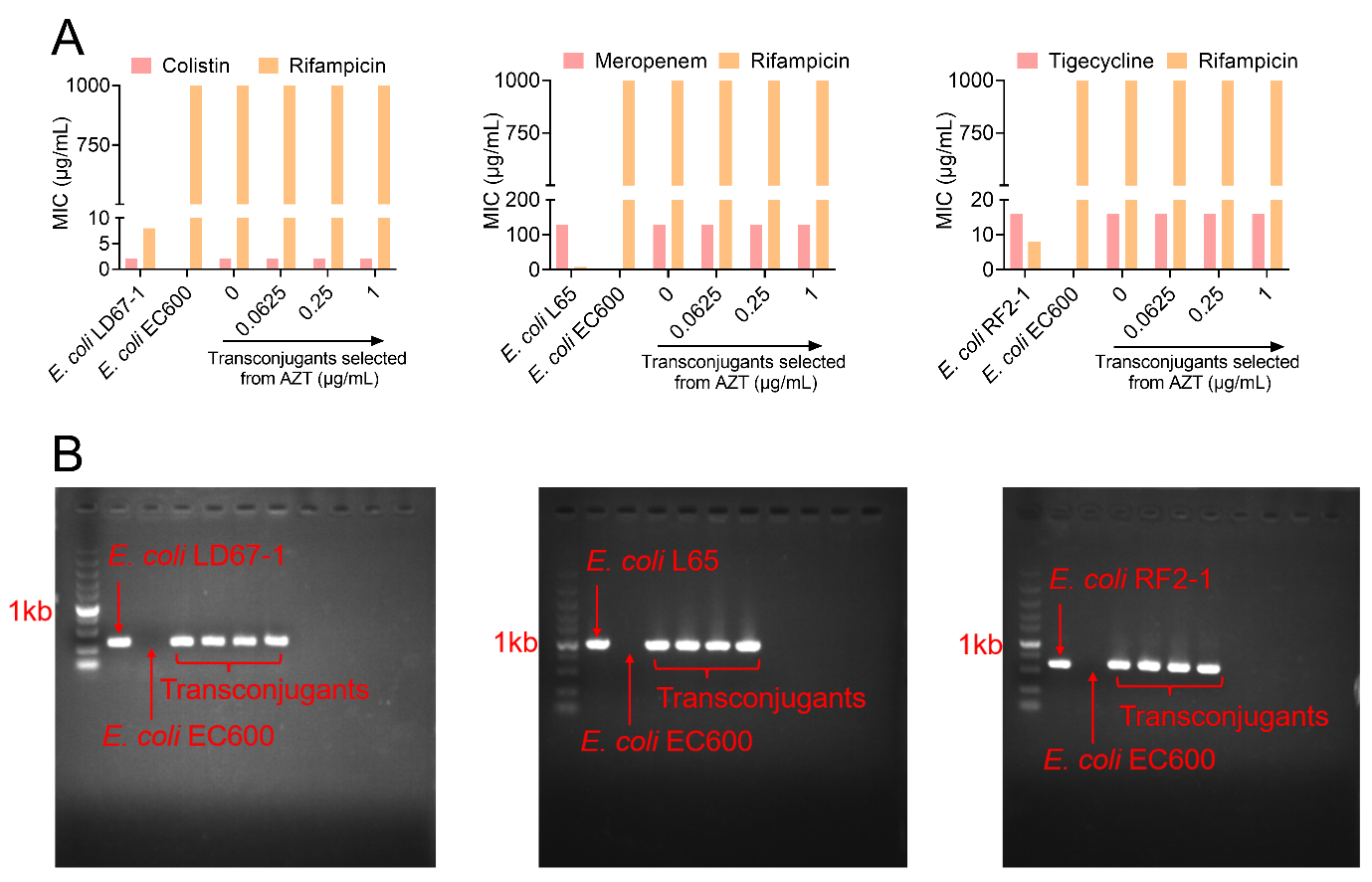


**Figure S3. MIC determination and PCR verification of donor, recipient and transconjugants bacteria under different concentrations of AZT.**

**(A)** MIC determination of colistin, meropenem, tigecycline and rifampicin was conducted according to EUCAST clinical breakpoints methods. **(B)** PCR verification of *mcr-1*, *bla*_NDM-5_ and *tet*(X4) primers and gel electrophoresis analysis were also performed to identify the accuracy of the transconjugants.





**Figure S4.** **Fold changes of conjugative transfer frequency of the engineered bacteria *E. coli* TOP10 carrying clinical *tet*(X4) plasmids after exposure to AZT.**

All data were presented as mean ± SD. Significant differences were evaluated by one-way ANOVA analysis and shown with ***P* < 0.01, ****P* < 0.001 and *****P* < 0.0001. n.s. not significant.





**Figure S5.** **Inhibitory effects of AZT on conjugation and chemical transformation.**

**(A and B)** The inhibitory effects of AZT on resistance plasmids from *P. vulgaris* **(A)** and *P. terrae* **(B)** to *E. coli* EC600 under 0.25 μg/mL AZT. **(C)** Conjugative transfer frequency of plasmid from *E. coli* RF2-1 to *K. pneumoniae* YZ6 after treatment with AZT. **(D-F)** The addition of AZT reduced the transformation frequency from pUC19 plasmid **(D),** pBAD plasmid **(E)** and pWM91 plasmid **(F)** to *E. coli* DH5ɑ, respectively.

Unpaired *t*-test between two groups or one-way ANOVA among multiple groups were used to calculate *P*-values (**P* < 0.05, ***P* < 0.01, ****P* < 0.001 and *****P* < 0.0001. n.s. not significant).





**Figure S6. Resistance development of *E. coli* to AZT after 10 days of serial passaging.**

The MIC values of *E. coli* RF2-1 **(A)** and *E. coli* RS3-1 **(B)** were determined at every 2 passages according to EUCAST clinical breakpoints methods. All MIC experiments were conducted with biological triplicates.
